# Reliability and validity of the Chinese version of the maternity-monitoring scale by parents

**DOI:** 10.1186/s12884-025-07880-x

**Published:** 2025-07-17

**Authors:** Tingzhi Chen, Mingyue Jia, Bingjie Zhang, Yongzhi Ji, Enshe Jiang

**Affiliations:** 1 Institute of Nursing and Health, Henan University, Kaifeng, 475004 China; 2 Department of Neurosurgery, The First Affiliated Hospital of Henan University, Kaifeng, 475004 China

**Keywords:** Postpartum depression, Parent rating scale, Cross-cultural adaptation, Reliability, Validity

## Abstract

**Background:**

Postpartum depression (PPD) affects women globally, yet self-report tools like the Edinburgh Postnatal Depression Scale (EPDS) face cultural and reporting biases. The parent-rated Maternity-monitoring Scale by Parents (MMSP), developed to address these gaps, was culturally adapted for China. This study validated the Chinese MMSP, offering a complementary tool to enhance PPD detection through familial observations.

**Methods:**

MMSP was translated and back translated using the modified Brislin translation model, and the Chinese test version of MMSP was formed after expert discussion, cultural debugging, and pre-investigation. From September to October 2024, convenience sampling was used to select 282 pregnant women and their parents from a Class A tertiary hospital in Henan Province to investigate the reliability and validity of the Chinese version of MMSP.

**Results:**

The Chinese version of MMSP consists of 15 items. The correlation coefficients between the scores of each item and the total score of the scale range from 0.873 to 0.935 (*P* < 0.05), and the critical ratios of each item range from 12.363 to 14.758 (*P* < 0.05). Exploratory factor analysis extracted one common factor with a cumulative variance contribution rate of 82.007%. The scale’s item-level content validity index (I-CVI) ranges from 0.83 to 1, and the scale’s average content validity index (S-CVI/Ave) is 0.93. Using the Chinese version of the Edinburgh Postnatal Depression Scale as the criterion, the criterion-related validity of MMSP is 0.972 (*P* < 0.05). The Cronbach’s α coefficient of the Chinese version of MMSP is 0.984, and the split-half reliability is 0.988.

**Conclusion:**

The Chinese version of MMSP has good reliability and validity, which can be used to effectively evaluate the depression status of postpartum women in clinical practice and help medical staff identify and intervene in potential depressive symptoms early.

## Background

Postpartum Depression (PPD) is a global health problem that affects women in different regions and cultural backgrounds [1]. The global average prevalence of postpartum depression is 19.18%, with significant differences between countries, and women in low - and middle-income countries are more susceptible to postpartum depression [2, 3]. This is due to the fact that postpartum physical recovery difficulties, sleep deprivation, breastfeeding difficulties, and lack of social support can all have an impact on the onset of postpartum depression [4, 5]. The World Health Organization (WHO) has recently highlighted the importance of integrating mental health into maternal and child health services and emphasized comprehensive care within routine maternal health services, including screening, prevention, and management of mental health disorders [6]. The guidelines advocate comprehensive care within routine maternal health services, including screening, prevention, and management of mental health disorders. The Edinburgh Postnatal Depression Scale (EPDS) is a widely used self-report scale to screen for the risk of postpartum depression. It was developed by Cox et al. in [7] to help medical professionals identify women who may be suffering from postpartum depression. However, because EPDS relies on individual self-reports and PPD is a disorder that is difficult for women to identify, many mothers believe that psychological difficulties in caring for their children should not be complained about. Therefore, EPDS may be affected by the individual’s cognitive, understanding and reporting tendency of symptoms, and some postpartum women may be unwilling or unable to accurately report their symptoms due to social expectations, shame, or misunderstanding of depression [8]. In China, the sensitivity and specificity of EPDS are 0.604 and 0.766, which indicates that EPDS ignores 40% of women with PPD [9]. Therefore, in addition to self-evaluation, PPD needs the evaluation of others. The Edinburgh Postnatal Depression Scale-Partner version (EPDS-P) is a Partner version of the EPDS, a screening tool used to assess depressive symptoms in postpartum women. The EPDS-P was developed by Moran and O’Hara in 2006 to evaluate postpartum women’s depressive symptoms through their partners, usually husbands [10]. This scale was designed with the idea that postpartum women’s partners could provide information about changes in maternal mood and behavior when postpartum women themselves were unwilling or unable to report their symptoms accurately because of societal expectations. However, strained or discordant partnerships may lead partners to have biased perceptions of postpartum women’s depressive symptoms. While parents of postpartum women are usually more sensitive to the emotional changes of their daughters. An extensive sample survey in China found that the caregivers of postpartum women are mainly borne by their mothers-in-law and mothers [11].

The Maternity-monitoring scale by parents (MMSP) was developed by Yoshiaki Ohashi’s team in Japan during the COVID-19 pandemic. During this period, the psychological stress of postpartum women increased significantly [12]. A national survey in Japan showed that the positive rate of EPDS increased to 28.7% during the pandemic, and this increase was attributed to anxiety caused by the pandemic, reduced social support, and limited direct communication between mother, child, and family [13]. To address this challenge, the MMSP scale was validated in two independent cohorts: a feasibility cohort (June 2020, during the first wave of the pandemic mitigation) and an emergency cohort (January 2021, during the Tokyo Declaration of Emergency). The feasibility cohort was designed to validate the psychometric properties of the scale in a relatively stable setting, whereas the emergency cohort was designed to assess its applicability in a high-stress setting. The scale, used by parents of postpartum women, provides a new tool to assess postpartum depression from the perspective of parents of postpartum women, helps to identify those with recessive PPD who may be overlooked by self-report scales, and takes into account the influence of cultural differences and family structure on the identification of postpartum depression, providing a new idea for the early identification of postpartum depression. This study aims to sinicize and verify the reliability and validity of MMSP in the context of Chinese culture, in order to provide more perspectives for the assessment of postpartum depression, enhance the ability to identify and intervene postpartum depression, and thus improve the mental health and quality of life of postpartum women.

## Data and methods

### Introduction to the scale

MMSP, developed by Ohashi Yoshiaki’s team in Japan [12], is a new screening tool for PPD based on the parental rating scale, which will be applied to the parents of postpartum women. MMSP consists of 15 items, a 5-point Likert scale was used, “Strongly agree” was 3 points, “Agree” was 2 points, “Disagree” was 1 point, “Strongly disagree” was 0 points, “Neither agree nor disagree” was 0 points. The total score is the sum of each item’s scores, a whole number between 0 and 45 for the individual. A higher score indicates a higher likelihood of PPD. Cronbach’s alpha coefficient of the MMSP scale had different values in the two cohorts: in the feasibility cohort, Cronbach’s alpha coefficient was 0.944. In the emergency cohort, Cronbach’s alpha coefficient was 0.961.

### Chinesization and cultural adaptation of the scale

#### Chinesization of the scale

This study was authorized by the original author via email to translate MMSP into Chinese using the standardized translation process proposed by Beaton et al. in [14], which is an improved version of the Brislin model. The process follows: ① Forward translation: Two native Chinese and bilingual professionals and English professionals independently translated the scale into two Chinese versions A1 and A2. ② Comprehensive translation: the Chinese version A1 and Chinese version A2 were discussed and revised by the researchers and their research groups to form the translated version A ③ back translation: Since the translated version must strictly respect the content of the original scale, two foreign teachers (native language is English, familiar with both Chinese and English and has taught English in China for 8 years or more) who have never been in contact with the scale and have no medical background are specially invited as translators. Two back translators should independently translate the back translation version A to form the back translation version B1 and the back translation version B2. After completion, B1 and B2 were compared by members of the research group and two back-translators, and the Chinese version of A was repeatedly discussed, revised, and back-translated until it was consistent with the original scale. The Chinese version of C was formed.

#### Cultural adjustment

Six experienced clinical experts were invited to conduct cultural debugging of the Chinese version of C. Selection criteria of experts: (1) master relevant professional knowledge and have more than 10 years of working experience; (2) Associate senior title or above; (3) Consent to participate in this study. Experts were invited to evaluate the items according to their professional knowledge and clinical work experience using the Likert 4-point scoring method based on readability, concept explanation, correlation between items and dimensions, language expression habits, and cultural background. After discussion and modification, the research group members sorted out the expert opinions and formed the Chinese version of D.

#### Pre-survey

Using the convenient sampling method, a total of 30 pregnant women and their parents who were admitted to a Class A tertiary hospital in Henan Province in September 2024 were selected for the test to evaluate whether the content of each item of the scale was clear and easy to understand and whether there were other suggestions and supplementary content. Inclusion criteria: (1) adult maternal women and their parents (2) able to fill in the required questionnaires independently or with the help of the researcher; (3) informed about diagnosis and consent to accept the investigators. Exclusion criteria: (1) patients with cognitive impairment or inability to express their thoughts accurately; (2) other psychiatric or central nervous system diseases; (3) physical weakness such as severe heart, lung, liver, kidney diseases or advanced malignant tumors; (4) Special circumstances: there may be extreme circumstances that affect the maternal psychological state (such as major family changes). All subjects gave informed consent and volunteered to participate in this study. Researchers should explain the purpose and significance of the study to the patients before the investigation. In the process of filling out the Chinese version of MMSP, the researchers need to ask the respondents whether the items are easy to understand, whether the language is clear, whether they know the content of the scale, explain patiently when they don’t understand the parts, and record the patients’ questions in time. After completing the questionnaire, the researcher asked the patients about their feelings and opinions on the scale and recorded the completion time. The language and content of MMSP were adjusted according to the subjects’ feedback. Finally, the Chinese version of MMSP was formed.

### The reliability and validity of the Chinese version of MMSP were tested

#### Respondents

From September to October 2024, convenience sampling was used to select pregnant women and their parents in a Class A tertiary hospital in Henan Province as the survey objects. The inclusion and exclusion criteria were the same as the pre-survey. The scale has 15 items. To ensure the reliability of factor analysis and improve the stability of the questionnaire structure, the sample size should be 5–10 times the number of items [15]. In addition, considering 20% invalid questionnaires, the sample size should be 94–188 cases. In this study, 282 questionnaires were distributed and 282 valid questionnaires were collected, with an effective recovery rate of 100%. This study has been approved by the Ethics Committee (Ethics number: HUSOM2024-526). All subjects voluntarily participated in the study and signed informed consent.

#### Research tools

The general information of the postpartum women and their parents, the Chinese version of the MMSP scale, and the Chinese version of the Edinburgh Postnatal Depression Scale were investigated by on-site collection. (1) General information questionnaire: self-designed by the researcher based on the literature review, including maternal age, number of past abortions, pregnancy, and births, the relationship between parents and the mother, and the frequency of contact with the mother. (2) Chinese version of MMSP: a total of 15 items, Likert 5-point scoring method was used, “strongly agree” was 3 points, “agree” was 2 points, “disagree” was 1 point, “strongly disagree” was 0 point, “neutral” was 0 point, the higher the score, the more likely the mother had PPD. (3) Chinese version of Edinburgh postnatal depression scale: To verify the validity of the MMSP scale in identifying the risk of postpartum depression and supplement the shortcomings of existing tools, the Chinese version of EPDS was selected as the criterion tool. EPDS was developed by Cox et al. [7], and the Chinese version of EPDS was translated and formulated by Lee et al. of the Chinese University of Hong Kong [16], which has been widely used in clinical practice. There were 10 items in EPDS; each item was scored from 0 to 3, of which the first and second items were reverse scores, and the remaining items were positive scores. The total score ranged from 0 to 30, and the higher the score, the greater the risk of postpartum depression. It should be noted that the EPDS is a self-rating scale, and the influence of family members and medical staff on the postpartum women should be avoided as much as possible during the filling process.

#### Data collection methods

With the consent of the relevant hospital departments, 4 research members formed a survey group to collect the questionnaires. Before the beginning of the survey, the purpose, the content of the questionnaire, and the requirements of filling in the questionnaire were uniformly trained to ensure that the four investigators used the exact instructions to explain to the respondents. Informed consent was required during the investigation, and the researchers conducted a face-to-face, one-to-one questionnaire survey. The participants and their parents were asked to fill out the questionnaire by themselves when they understood the content of the scale. After completing the questionnaire, the investigators immediately checked its completeness.

#### Statistical methods

SPSS 27.0 software was used to analyze the data. Qualitative data were expressed as frequency and percentage (%). The quantitative data by normal distribution were expressed as mean ± standard deviation ($\overline{\mathrm x}\pm\mathrm s$). The item-level content validity index (I-CVI) and the scale-average content validity index (S-CVI/Ave) were used to evaluate the scale’s content validity. The Spearman correlation coefficient method was used to test the correlation between the total scores of the Chinese version of MMSP and the total scores of the Chinese version of EPDS. Exploratory factor analysis was used to evaluate the construct validity, and Cronbach’s α coefficient and split-half coefficient were used to assess reliability. *P* < 0.05 was considered statistically significant.

## Results

### The Chinese and cross-cultural adaptation of MMSP

According to the cross-cultural debugging process of Beaton et al., forward translation, comprehensive translation, and back translation of MMSP were carried out, and the Chinese version C was formed through repeated comparison [14]. In the expert review section, two experts in obstetric clinical medicine, two in obstetric nursing, one in medical English, and one in psychiatry and psychology were invited to form an expert panel for discussion. The age of the experts was (45.67 ± 7.53) years. 4 senior and 2 associate seniors; The average working years were (18.50 ± 7.23) years. The specific modifications of the scale are as follows: Change item 3 from"She doesn’t seem to sleep extremely"to"She doesn’t seem to be getting enough sleep."item 4 from"She doesn’t seem to enjoy life."to"She can’t seem to enjoy life."item 6 from"She is badly groomed.” to"Her appearance is not good."item 9 from"She is irritated that she can’t care her baby and household chores."to"She is angry at not being able to take care of the children and keep house."Item 10 from, “She says I wish to disappear."to"She doesn’t want to see anyone."item 12 from” She has lost her expression on everything."to"She has become numb to everything."item 15 from “She tears” to “She shed tears”. In the pre-survey process, the parents of postpartum women participating in the study said they could understand the items of the Chinese version of MMSP and did not put forward any questions or suggestions (see Table 1).

**Table 1:** The Chinese-maternity-monitoring scale by parents

Items	English Version	Chinese Version
MMSP01	She seems depressed and gloomy.	她看起来神情沮丧
MMSP02	She says I don’t think my baby dear.	她认为她和孩子关系不亲近
MMSP03	She doesn’t seem to sleep extremely.	她看起来睡眠不足
MMSP04	She doesn’t seem to enjoy life.	她似乎无法享受生活
MMSP05	She doesn’t seem to be able to focus on caring for her baby and household chores.	她似乎无法集中精力照顾孩子和做家务
MMSP06	She is badly groomed.	她仪容很不整洁
MMSP07	She says I’m so tired of parenting.	她说我厌倦了为人父母
MMSP08	She says something that blames herself.	她说了些责备自己的话
MMSP09	She is irritated that she can’t care her baby and household chores.	她为不能照顾孩子和收拾家务而气恼
MMSP10	She says I wish to disappear.	她不愿见任何人
MMSP11	She seems frustrated by trivial things.	她为一些琐碎小事而沮丧
MMSP12	She has lost her expression on everything.	她对一切都变得麻木
MMSP13	She says about worries and anxieties.	她提及担心和焦虑
MMSP14	She doesn’t seem to be happy with her Baby’s growth.	她对孩子的成长没有感到高兴
MMSP15	She tears.	她流泪了

### Demographic characteristics of participants

A total of 282 parents were included, including 97 males (34.4%) and 185 females (65.6%). Among them, 54 cases (19.1%) played the role of father, 95 cases (33.7%) played the role of mother, 43 cases (15.2%) played the role of father-in-law, and 90 cases (32.0%) played the role of mother-in-law. The age ranged from 40 to 65 (55.26 ± 5.83) years (see Table 2).

**Table 2:** Demographic characteristics of participants (*n* = 282)

Variables	*N* (%) or Mean (SD)
Age	55.26 (5.83)
Sex	
Male	97 (34.4)
Female	185 (65.6)
Role	
Father	54 (19.1)
Mother	95 (33.7)
Father-in-law	43 (15.2)
Mother-in-law	90 (32)

*SD* Standard deviation

### Scale item analysis results

**Fig. 1:**
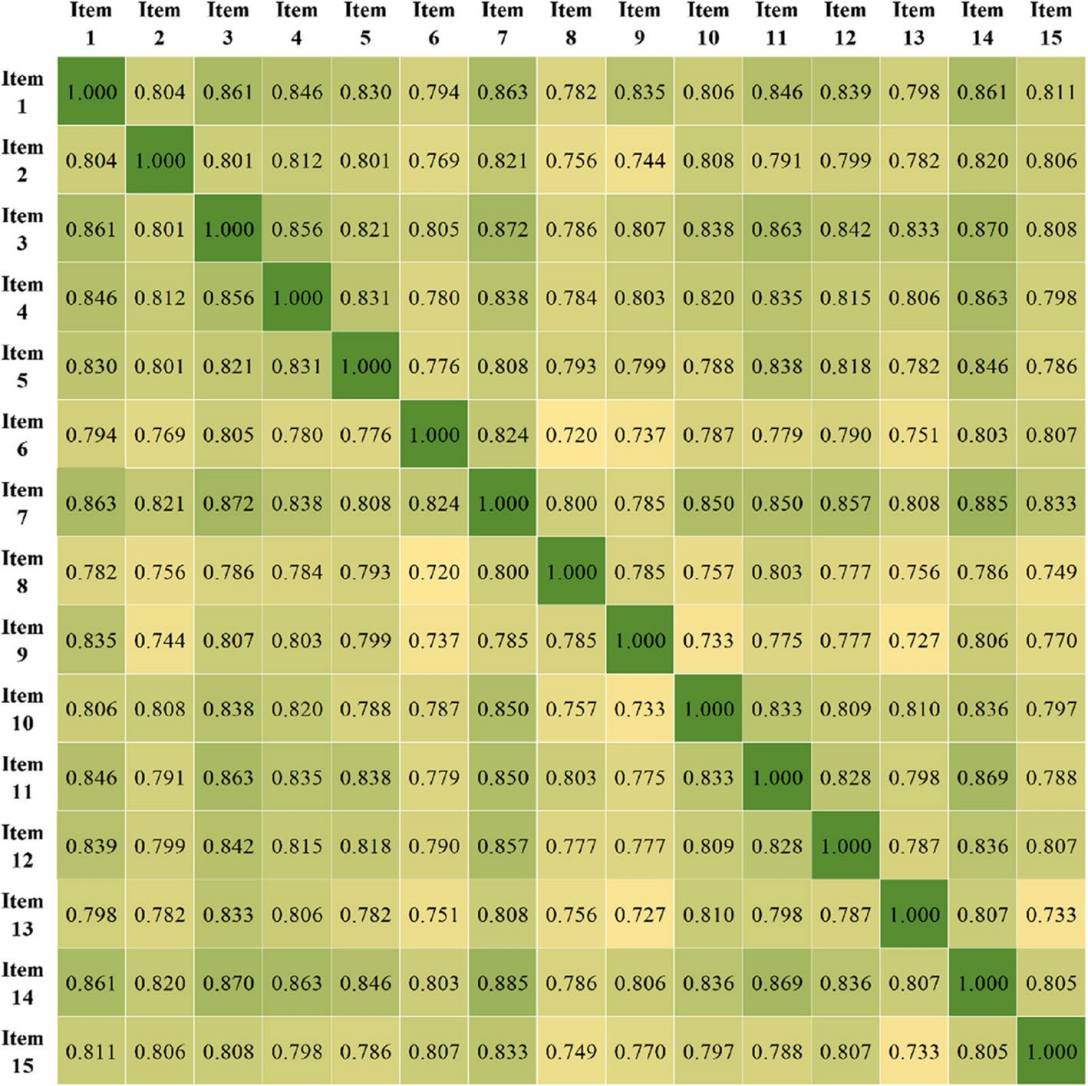
Inter-item correlation coefficients between all MMSP items

The critical ratio (C.R.) method can be used to assess discrimination against items. The total scores of 282 questionnaires were ranked from high to low, and the top 27% of the samples were classified as the high-score group, while the bottom 27% were classified as the low-score group. Two independent sample t-tests were conducted on the scores of each item in the high and low-score groups, and items with a C.R. value > 3 and *P* < 0.05 were retained [17]. The item analysis results showed that the C.R. values of the Chinese version of the MMSP ranged from 12.363 to 14.758, all of which were greater than 3, and the significance levels of each item’s C.R. values were all less than 0.05. This indicates that the discrimination of the items was good. The item-total correlation analysis was used to assess the discrimination of the items. The correlation coefficients between each item of the MMSP scale and the total score were calculated, and items with a correlation coefficient (R-value) > 0.4 and *P* < 0.05 were retained [18]. The correlation analysis showed that the correlation coefficients between each item and the total score of the MMSP scale were 0.873 to 0.935 (Table 3), and the correlation coefficients between each item were highly correlated (Fig. 1) and statistically significant (*P* < 0.05), so all items were retained for subsequent analysis.

**Table 3:** Exploratory factor analysis results of the Chinese MMSP scale

Items	Critical ratio method		Correlation coefficient method		Standardized Factor loading	Whether to keep it
	C.*R*. value	*P* value	Coefficient of correlation	*P* value		
1.她看起来神情沮丧/She looks depressed.	14.758	<0.05	0.926	<0.05	0.926	Keep
2.她认为她和孩子关系不亲近/She doesn’t think she has a close relationship with her child.	13.166	<0.05	0.892	<0.05	0.892	Keep
3.她看起来睡眠不足/She doesn’t seem to be getting enough sleep.	13.500	<0.05	0.933	<0.05	0.933	Keep
4.她似乎无法享受生活/She can’t seem to enjoy life.	14.688	<0.05	0.920	<0.05	0.920	Keep
5.她似乎无法集中精力照顾孩子和做家务/She seems unable to concentrate on child care and housework.	14.041	<0.05	0.907	<0.05	0.907	Keep
6.她仪容很不整洁/Her appearance is not good.	13.399	<0.05	0.877	<0.05	0.877	Keep
7.她说我厌倦了为人父母/She says I am tired of being a parent.	13.166	<0.05	0.935	<0.05	0.935	Keep
8.她说了些责备自己的话/She says something to blame herself.	13.726	<0.05	0.873	<0.05	0.871	Keep
9.她为不能照顾孩子和收拾家务而气恼/She is angry at not being able to take care of the children and keep house.	14.384	<0.05	0.875	<0.05	0.874	Keep
10.她不愿意见任何人/She doesn’t want to see anyone.	13.914	<0.05	0.903	<0.05	0.904	Keep
11.她为一些琐碎小事而沮丧/She is upset about trifles.	13.166	<0.05	0.920	<0.05	0.921	Keep
12.她对一切都变得麻木/She has become numb to everything.	13.500	<0.05	0.912	<0.05	0.912	Keep
13.她提及担心和焦虑/She mentioned worry and anxiety.	12.569	<0.05	0.883	<0.05	0.882	Keep
14.她对孩子的成长没有感到高兴/She is not happy with the growth of her child.	13.021	<0.05	0.935	<0.05	0.935	Keep
15.她流泪了/She shed tears.	12.363	<0.05	0.890	<0.05	0.890	Keep

### Validity

#### Content validity

This study invited six experts to conduct a panel discussion on the Chinese version of MMSP to evaluate the scale’s content validity. The experts were asked to assess the association (or representativeness) of each item of the evaluation questionnaire with the corresponding content dimension using a 4-point Likert scale. A score of 1 indicated no correlation, 2 indicated weak correlation, 3 indicated strong correlation, and 4 indicated robust correlation. The I-CVI and S-CVI/Ave were calculated, respectively. When the I-CVI of the scale items was ≥ 0.78, and the S-CVI/Ave reached 0.9, it indicated that the scale’s content validity was good [19]. According to the expert evaluation results, the I-CVI of the Chinese version of MMSP was 0.83 to 1.00, and the S-CVI/Ave was 0.93.

#### Validity of construct

**Fig. 2:**
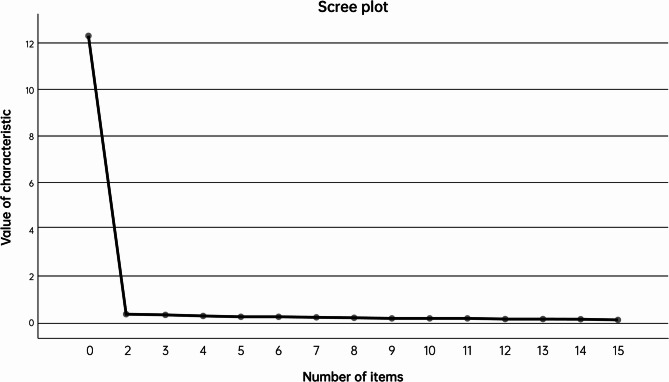
Scree plot of factor analysis of the Chinese version of the maternity-monitoring scale by parents

Exploratory factor analysis was used to evaluate the structural validity of the Chinese version of MMSP. Firstly, the KMO test and Bartlett’s sphericity test were conducted. If the KMO value was greater than 0.8 and the χ² value of Bartlett’s sphericity test reached a significant level (*P* < 0.05), it indicated that the scale was suitable for exploratory factor analysis [20]. The item loading value on a factor > 0.4 was used as the criterion for factor attribution, and the double loading items were deleted [21]. The factor loading of the Chinese version of MMSP was all more than 0.4. The KMO test value of the Chinese version of the MMSP scale was 0.984, with a good partial correlation. Bartlett’s sphericity test showed that the approximate x^2^ value was 5906.899, *P* < 0.05, indicating that it was suitable for exploratory factor analysis. Principal component analysis was used to extract common factors and obtain the initial loading matrix. Then, the orthogonal rotation method was used to obtain the final factor loading matrix [22]. A total of one common factor was extracted. The scree plot (Fig. 2) suggested extracting one factor with strong loading, indicating that the Chinese version of MMSP was a unidimensional scale, and its structure was consistent with the original scale. The cumulative variance contribution rate was 82.007%. The results showed that 15 items could reflect the theme of postpartum depression, so all items were retained. See Table 3.

#### Criterion-related validity

Spearman correlation analysis was used to test the correlation between the total score of the Chinese version of MMSP and the total score of the Chinese version of EPDS. A correlation coefficient between 0.400 and 0.800 indicates a moderate correlation, and a correlation coefficient > 0.800 indicates a high correlation. The correlation coefficient between the Chinese version of MMSP and the total score of the Chinese version of EPDS was 0.972(*P* < 0.05).

### Reliability

Cronbach’s α coefficient was used to evaluate the internal consistency of the Chinese version of MMSP. The split-half coefficient was calculated using the parity split-half method, in which each scale item was divided into two halves. Then, the correlation coefficient of the scores of the two parts was calculated. Cronbach’s α and split-half coefficients were > 0.7 as the standard to judge the scale’s reliability [23]. The Cronbach’s coefficient of the Chinese version of MMSP was 0.984, and the split-half reliability coefficient was 0.988.

## Discussion

### The Chinese version of MMSP is readable and understandable

This study followed Beaton et al.‘s translation process to translate, back-translate, cross-culturally adapt, and pre-test the MMSP [14]. The Chinese version of MMSP, after localization, includes 15 items. No conflicts related to language content were found during the forward and backward translation processes. During the expert consultation process, six experts from different fields discussed and adjusted the items’ readability, relevance, language expression habits, and cultural background to align the Chinese MMSP with the local context and cultural characteristics. During the pre-test process, all participants indicated that they could understand the content of the items in the Chinese version of MMSP and did not raise any questions or suggestions. Finally, this study completed the reliability and validity tests in postpartum women and their parents. The items of the Chinese version of MMSP are concise, clear, and easy to understand, showing good reliability and validity, and can accurately reflect the depression status and related factors of postpartum women. This provides medical personnel with an effective tool to promote the long-term mental health of postpartum women and provides a basis for managers to formulate mental health promotion strategies and evaluate the application effect of mental health intervention strategies from the perspective of postpartum women and their families, which has high clinical application value.

### The Chinese version of MMSP has good validity

Six experts from different fields with rich clinical experience and high academic levels participated in the content validity evaluation of this study. After the discussion, the experts evaluated the content validity of the scale. The results showed that the 1-CVI ranged from 0.83 to 1.0, and the S-CV/Ave was 0.93, indicating that the scale’s content validity was good and could effectively measure the psychological status of postpartum women [19]. Exploratory factor analysis extracted one common factor, consistent with the original scale, and the cumulative variance contribution rate was 82.01%, indicating that the scale could measure the same content, namely, postpartum depression. Taking the Chinese version of EPDS as the criterion, the criterion-related validity was 0.972 (*P* < 0.05), indicating that the criterion-related validity of the questionnaire was good. In the study by Ohashi Yoshiaki et al., the criterion-related validity of the feasibility cohort and the emergency cohort was 0.581 and 0.518 [12]. which were different from the results of this study. This might be because the Japanese study was conducted during the COVID-19 pandemic, where social isolation and anxiety could have limited parents’ observational abilities. The Chinese study was carried out in 2024, when the pandemic had subsided and social order was relatively stable, reducing the interference of external pressures on data quality. Secondly, during pregnancy in China, the husband usually takes on the responsibility of providing for the family [24]. After childbirth, as in many other Asian countries, Chinese women typically have a “postpartum confinement” to recover from the physical strain of giving birth [25]. During this time, it is usually the mother or mother-in-law of the new mother who stays with her, providing postpartum care, closely observing her daily behavior and emotional changes. This high-frequency interaction may have enhanced parents’ sensitivity to depressive symptoms and the accuracy of their reports. In contrast, in the Japanese study, the rate of parents’ neglect of PPD symptoms significantly increased during the pandemic (from 33–50%) [12]. Additionally, in recent years, there has been a significant increase in China’s social attention to mental health, with the government and media actively promoting mental health education, reducing related stigmatization. This has made family members more willing to acknowledge and report the emotional problems of new mothers. Item analysis was conducted using the critical ratio method and the item-total correlation analysis method. The critical ratio method showed that all scale items discriminated well between high and low-score groups. The item-total correlation analysis demonstrated strong correlations between individual items and the total MMSP scores. These items reflect key constructs of postpartum depression originally proposed in the Japanese MMSP study, including mood responsiveness (item 3), sleep (item 4), ability to concentrate (item 5), fatigue (item 6), and worthlessness (item 9), etc [12]. The research further validate the ability of the scale to capture core symptoms of PPD through parental observation. The item analysis results also proved, to some extent, the validity of the Chinese version of MMSP.

### The Chinese version of MMSP has good reliability

Reliability refers to the consistency and accuracy of the measurement results obtained using a specific tool. When evaluating the reliability of the Chinese version of MMSP, Cronbach’s α and split-half reliability were used to assess its internal consistency [26]. Cronbach’s coefficient of this study was 0.984. The Cronbach’s α coefficient of the original scale had different values in two cohorts: 0.944 in the feasibility cohort and 0.961 in the emergency cohort. This indicates that the internal consistency of the Chinese version of the scale is good. Split-half reliability reduces the influence of items on reliability estimation by splitting the scale, especially when there are many items in the scale. The split-half reliability of the Chinese version of MMSP was 0.988. Overall, this indicates that the Chinese scale version is reliable.

### The Chinese version of MMSP is suitable for the assessment of postpartum depression

Postpartum depression is a psychological issue that occurs in women after giving birth, typically characterized by persistent low mood, depressive symptoms, or depressive episodes. Different from the common “postpartum blues” or “baby blues”, postpartum depression has more severe symptoms and lasts longer, usually presenting within 6 weeks to a year after childbirth, and requires timely attention and treatment. A prospective, multicenter cohort study shows that postpartum blues and their severity are related to PPD. Early identification and intervention can improve the long-term outcome of the mother-infant relationship and reduce the incidence of PPD [27]. The Chinese version of the MMSP scale, as a parent-rated scale designed explicitly for postpartum depression screening, takes into account that women with postpartum depression may have difficulty self-identifying depressive symptoms and that cultural factors may prevent them from expressing their psychological distress. Assessing the potential depressive risk of postpartum mothers through their parents helps identify women with latent PPD.

## Limitations

Although the Chinese version of MMSP can effectively assess postpartum depression in women, it still has some limitations. The sample source of this study is single, and the reliability and validity verification were only completed in one hospital. This may be affected by regional culture and medical resource differences, which limit the universality of the conclusion. In addition, in the Chinese cultural background, the proportion of female caregivers (mother-in-law or mother) among the research subjects is relatively high, which leads to a bias towards the female perspective in the scoring. This cultural restriction may inevitably introduce some biases. At the same time, with the development of society and the improvement of medical conditions, more and more new mothers choose to spend this period in a “confinement center” without the company of their parents. This change may have a certain impact on the research results, because our research subjects are the parents accompanying the new mothers, which may not fully represent the situation of all new mothers. In the future, it is necessary to expand the sample coverage, balance the gender ratio, and include heterogeneous groups from multiple centers and multiple levels of medical institutions to further verify the wide applicability of the scale.

## Conclusions

This study conducted the Chinese version of the MMSP’s translation and initial validation. The results indicated that the Chinese version of MMSP has good reliability and validity, retains the characteristics of the original scale, and can be used for the assessment and screening of PPD in postpartum mothers. The Chinese version of MMSP is a one-dimensional scale with 15 items, using a 5-point Likert scale. It is simple to measure and easy to understand. It can provide a reference for medical personnel to identify early, formulate personalized intervention measures, and improve the disease awareness level of postpartum women.

## Data Availability

The datasets used and/or analyzed during the current study available from the corresponding author on reasonable request.

## Abbreviations

WHO: World Health Organization
PPD: Postpartum depression
EPDS: Edinburgh Postnatal Depression Scale
EPDS-P: Edinburgh Postnatal Depression Scale-Partner version
MMSP: Maternity-Monitoring Scale by Parents
I-CVI: Item-Content Validity Index
S-CVI: Scale-Content Validity Index
C.R.: Critical ratio
KMO: Kaiser-Meyer-Olkin
